# Investigation on the Structural and Photocatalytic Performance of Oxygen-Vacancy-Enriched SnO_2_-CeO_2_ Heterostructures

**DOI:** 10.3390/ijms242015446

**Published:** 2023-10-22

**Authors:** Daixiong Yang, Yangwen Xia, Ling Zhang, Jiawei Liu, Xiaodong Zhu, Wei Feng

**Affiliations:** School of Mechanical Engineering, Chengdu University, Chengdu 610106, China; yangdaixiong1998@163.com (D.Y.); x1278704108@163.com (Y.X.); jiawei081999@163.com (J.L.)

**Keywords:** CeO_2_, SnO_2_ coupling, oxygen vacancy, photocatalytic performance

## Abstract

In this study, pure CeO_2_ and oxygen-vacancy-enriched SnO_2_-CeO_2_ composite materials were prepared using the sol–gel method, and their microstructures and photocatalytic properties were investigated. The results indicate that SnO_2_ coupling promotes the separation and transfer of photogenerated electrons and holes and suppresses their recombination. The 50% SnO_2_-CeO_2_ composite material exhibited a decreased specific surface area compared to pure CeO_2_ but significantly increased oxygen vacancy content, demonstrating the highest photogenerated charge separation efficiency and the best photocatalytic performance. After 120 min of illumination, the degradation degree of MB by the 50% SnO_2_-CeO_2_ composite material increased from 28.8% for pure CeO_2_ to 90.8%, and the first-order reaction rate constant increased from 0.002 min^−1^ to 0.019 min^−1^.

## 1. Introduction

Catalytic technology powered by light, occurring through a process known as photocatalysis, has found extensive applications in water pollution treatment. Cerium dioxide (CeO_2_)-based photocatalytic materials have garnered significant attention due to their high catalytic activity, strong stability, cost-effectiveness, and harmlessness to human health [1,2,3,4]. However, pure CeO_2_ possesses a relatively wide bandgap, which limits its ability to make use of solar energy. Additionally, pure CeO_2_ suffers from a rapid recombination of photo-generated electrons and holes, leading to low quantum efficiency. Therefore, the key to enhancing CeO_2_ photocatalytic performance lies in modifying CeO_2_ to improve solar light utilization and reduce the recombination rate of photogenerated electrons and holes [5,6,7,8].

Commonly employed methods for CeO_2_ modification include heterojunction construction and defect engineering [9,10,11,12]. Coupling CeO_2_ with another semiconductor to create a semiconductor heterojunction has proven effective in enhancing CeO_2_’s photocatalytic performance. The differing positions of the valence and conduction bands between the two semiconductors not only expedite the transfer of photogenerated charges but also reduce the bandgap width of the photocatalytic material, thereby increasing its light absorption range. Semiconductors such as CuO, g-C_3_N_4_, and Co_3_O_4_ are often used to form semiconductor heterojunctions with CeO_2_ [13,14,15]. Zhang et al. [13] prepared CuO@CeO_2_ composite materials via a template-assisted method. The materials exhibited a synergistic effect on the adsorption and activity of ammonia-containing substances, and the synergistic effect became more pronounced with an increasing CuO content. Zhao et al. [14] synthesized g-C_3_N_4_-CeO_2_ composite materials using an in situ pyrolysis method, significantly reducing the recombination rate of photogenerated electrons and holes compared to pure CeO_2_ and g-C_3_N_4_. After 2.5 h of the photocatalytic degradation of BPA, the degradation degree of the composite material reached 94.1%. On the other hand, increasing the concentration of oxygen vacancies (OVs) is a commonly used approach to enhance the physical and chemical properties of semiconductor photocatalysts. OVs not only facilitate the separation of photogenerated electrons and holes and expand the light absorption range but also provide more reaction sites [16,17,18]. Zhang et al. [17] prepared CeO_2_ modified with oxygen-vacancy-rich Prussian Blue (PB), resulting in a significant increase in Ce^3+^ content in the modified CeO_2_, indicating an enhanced quantity of oxygen vacancies. After 60 min of reaction, the PB-modified CeO_2_ exhibited a degradation degree for norfloxacin that increased from 5.84% to 82.05%. Kong et al. [18] prepared CeO_2_ through a template method and prepared oxygen-vacancy-rich CeO_2_ via oxidation-reduction and a steam treatment. The presence of oxygen vacancies increased the light absorption of CeO_2_ and reduced the recombination rate of the photogenerated electrons and holes. After 25 h of photocatalysis under 200 °C conditions, there was no significant decrease in activity.

In this study, we synthesized oxygen-vacancy-rich SnO_2_-CeO_2_ composite materials with varying Sn/Ce molar ratios using the sol–gel method. We evaluated the photocatalytic performance of the samples using methylene blue (MB) as the target pollutant and discussed the impact of bandgap width, separation, transfer, and recombination of photogenerated electrons and holes on photocatalytic performance. Furthermore, we investigated the photocatalytic mechanism for organic pollutant removal through reactive species experiments.

## 2. Results and Discussion

**Figure 1:**
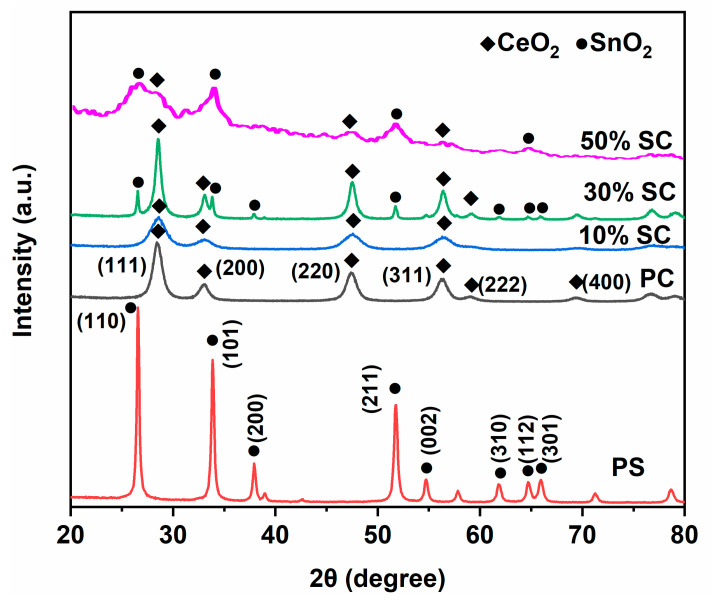
XRD patterns of samples.

Figure 1 shows the XRD (X-ray diffraction) patterns of the samples. In the XRD pattern of pure CeO_2_, diffraction peaks appeared at 2θ values of 28.6°, 33.2°, 47.5°, 57.4°, 69.6°, and 79.1°, corresponding to the (111), (200), (220), (222), (400), and (420) crystal planes of CeO_2_, respectively [7,19,20]. For pure SnO_2_, diffraction peaks were observed at 2θ values of 26.7°, 33.8°, 37.9°, 51.8°, 61.1°, 64.6°, and 65.1°, corresponding to the (110), (101), (200), (211), (002), (310), (112), and (301) crystal planes of SnO_2_, respectively [14,21,22]. When the amount of Sn added to the CeO_2_ sample exceeded 30%, diffraction peaks of SnO_2_ began to appear, indicating that the sample was a two-phase composite material of CeO_2_ and SnO_2_. In the 10% SC sample, no diffraction peaks of SnO_2_ were observed, possibly because the amount of Sn added was too low, and SnO_2_ was present in the sample In the form of dispersed small particles, making It difficult to detect the characteristic peaks of SnO_2_ [23]. The absence of any other diffraction peaks in all the samples indicates that the prepared samples had relatively high purity. The crystallinity of the 50% SC sample was the poorest among all the samples; notably, appropriate surface defects can enhance the photocatalytic performance of a sample [24].

**Figure 2:**
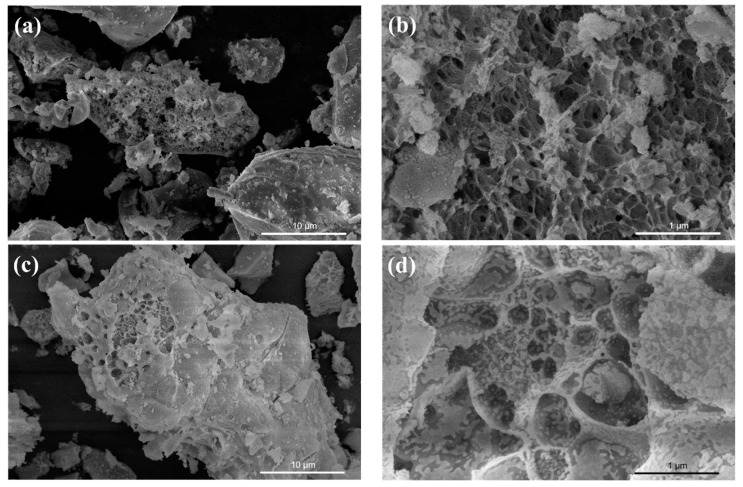

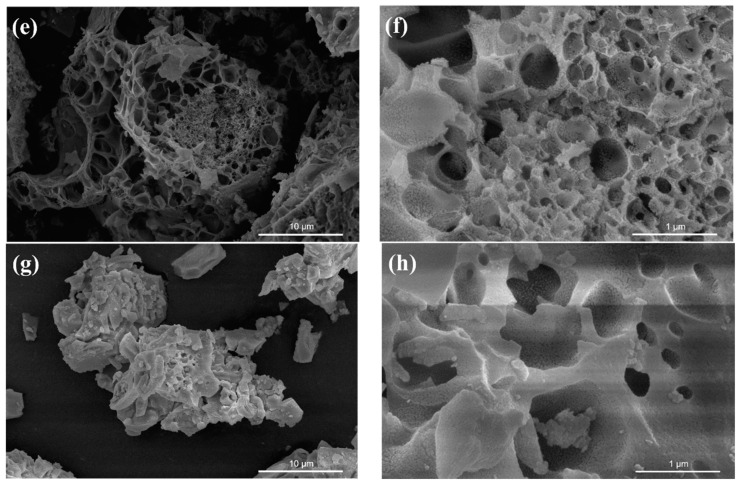
SEM images of PC (**a**,**b**), 10% SC (**c**,**d**), 30% SC (**e**,**f**), and 50% SC (**g**,**h**).

Figure 2 displays SEM images of the samples. In the figure, it can be observed that pure CeO_2_ exhibits a porous structure with evident aggregation. The aggregation phenomenon was not alleviated in the case of SnO_2_ coupling. Notably, the composite samples have larger pore diameters compared to pure CeO_2_.

**Figure 3:**
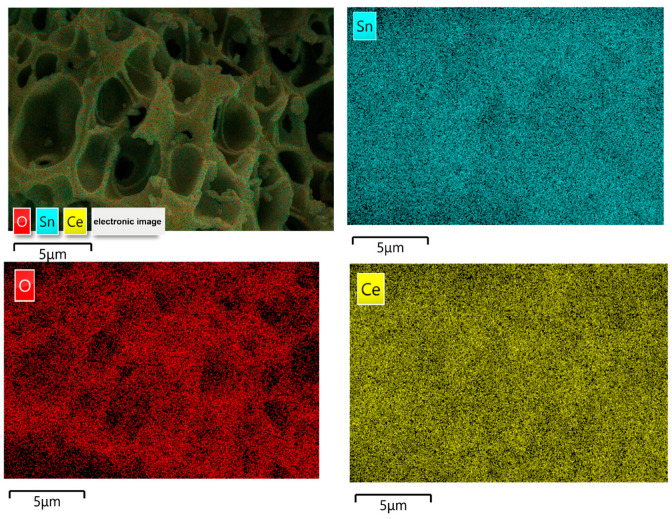

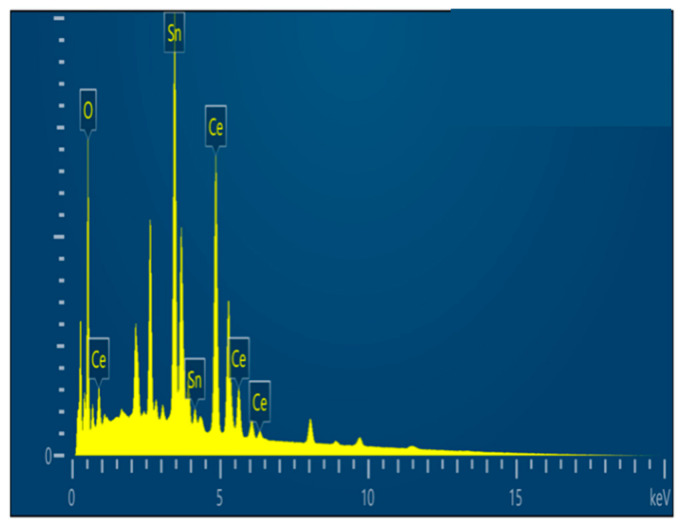
Element distribution mappings and EDS spectra of 50% SC.

Figure 3 shows the element distribution mappings and EDS (energy-dispersive X-ray spectroscopy) spectra of 50% SC, confirming the presence of O, Sn, and Ce in the samples, distributed relatively uniformly in the matrix.

**Figure 4:**
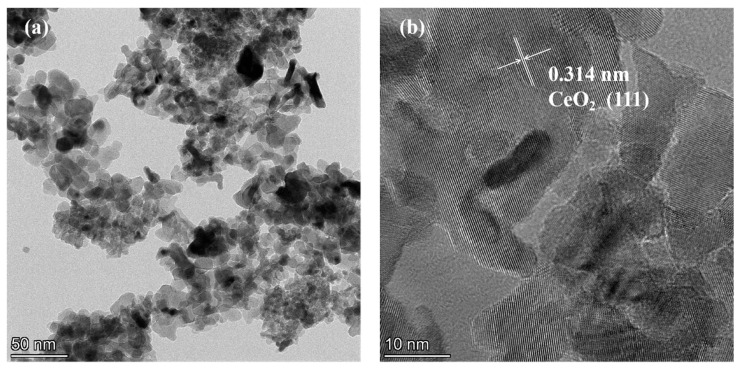

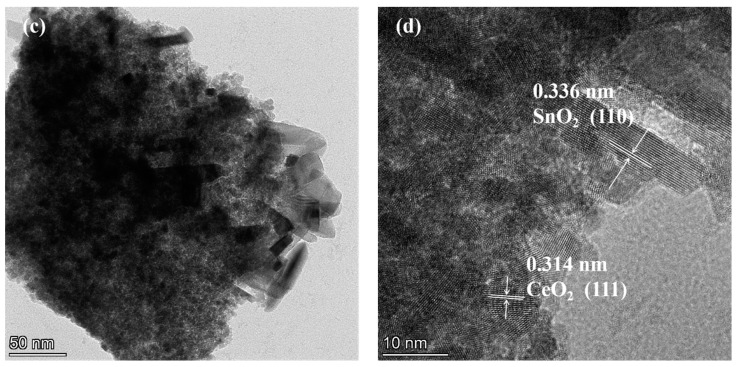
TEM and HRTEM images of PC (**a**,**b**) and 50% SC (**c**,**d**).

Figure 4 presents TEM (transmission electron microscopy) images of the samples. In Figure 4a, the sample consists of numerous particle-shaped pieces of CeO_2_ and some rod-shaped pieces of CeO_2_, displaying clear lattice fringes, indicating good crystallinity. The labeled interplanar spacing of 0.314 nm in Figure 4b corresponds to the (111) crystal plane of CeO_2_ [25,26]. In Figure 4c, most of the samples appear rod-shaped, and the labeled interplanar spacings of 0.314 nm and 0.336 nm correspond to the (111) crystal plane of CeO_2_ and the (110) crystal plane of SnO_2_ [19,27,28], respectively, confirming that the 50% SC sample is a CeO_2_-SnO_2_ composite material, in accordance with the XRD test results.

**Figure 5:**
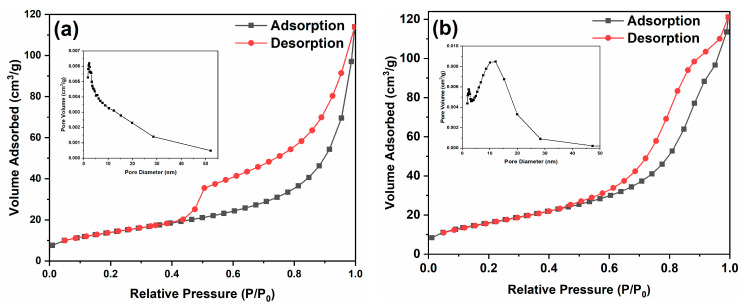

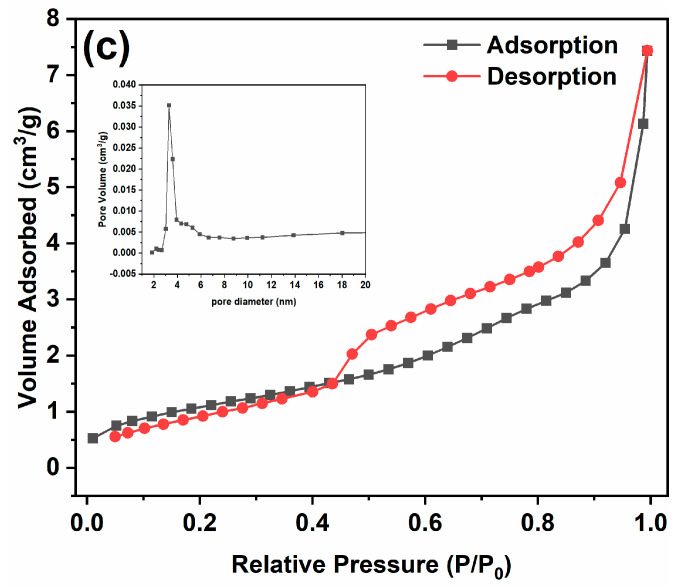
N_2_ adsorption desorption isotherms of the samples: PC (**a**), 30% SC (**b**), and 50% SC (**c**).

Figure 5 presents the N_2_ adsorption–desorption isotherms of the samples. In Figure 5a, it is evident that the pore size of PC mainly centers around 4.7 nm, while the pore sizes of the 30% SC and 50% SC composite materials predominantly center around 14.5 nm and 8.1 nm, respectively, indicating that SnO_2_ coupling was able to increase the pore size of the samples. The specific surface area of PC is 51.2 m^2^/g, while that of the 30% SC and 50% SC samples is 60.7 m^2^/g and 4.1 m^2^/g, respectively. The specific surface area of the 30% SC sample increased compared to PC, but that of the 50% SC sample decreased, possibly due to the lower crystallinity and significant aggregation in the 50% SC sample, resulting in a relatively lower specific surface area compared to PC.

**Figure 6:**
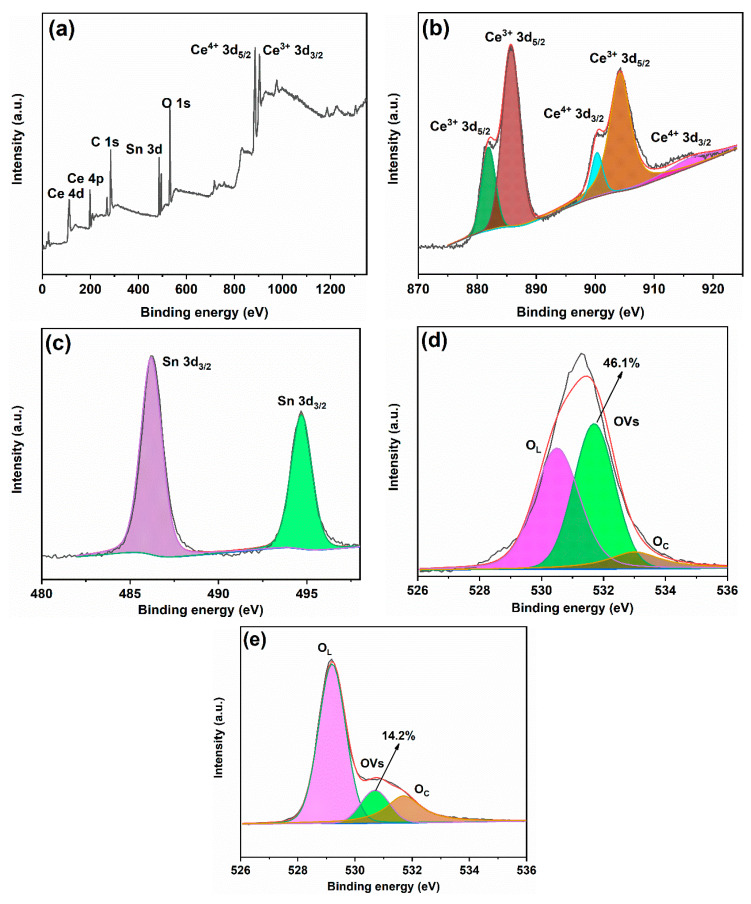
XPS spectra of 50% SC (**a**–**d**) and 30% SC (**e**).

Figure 6 presents the XPS (X-ray photoelectron spectroscopy) spectra of the samples. Figure 6a shows the survey spectrum of 50% SC, which reveals peaks corresponding to C, Ce, O, and Sn in the sample. Figure 6b displays the Ce 3d spectrum of 50% SC, showing five distinct peaks at binding energies of 881.9 eV, 885.7 eV, 904.3 eV, 900.3 eV, and 916.3 eV. The peaks at 881.9 eV, 885.7 eV, and 904.3 eV correspond to Ce^3+^ states, while the peaks at 900.3 eV and 916.3 eV belong to Ce^4+^ states [1,29,30]. Generally, CeO_2_ is a fluorite-type oxide with Ce^4+^ occupying its face-centered cubic lattice in its crystal cell and O^2−^ in tetrahedral positions. Thus, each Ce^4+^ is surrounded by eight O_2_^−^s, with O_2_^−^ primarily coordinating with Ce^4+^. The presence of Ce^3+^ indicates the generation of oxygen vacancies in the material, which is consistent with the photoluminescence (PL) description [1,24,31]. As shown in Figure 6c, the Sn 3D spectrum of 50% SC exhibits peaks at binding energies of 486.4 eV and 494.9 eV, corresponding to Sn 3d_5/2_ and Sn 3d_3/2_, respectively, indicating Sn in the +4 state [32,33]. Figure 6d displays the O 1s peaks of 50% SC, with peaks at binding energies of 531.7 eV, 530.5 eV, and 533.0 eV corresponding to lattice oxygen (OL), oxygen vacancies (OVs), and chemisorbed oxygen-containing species (OC) [34,35,36]. The content of oxygen vacancies can be estimated using the following formula: S_OVs_/S. Here, S represents the total area of the O 1s region, and S_OVs_ represents the peak area of oxygen vacancies on the sample surface [37,38]. Through calculation, the oxygen vacancy content was found to be 46.1%. Figure 6e shows the O 1s peaks of 30% SC, and the calculated oxygen vacancy content is 14.2%. The oxygen vacancy content significantly increased in 50% SC compared to 30% SC. In general, oxygen vacancies can serve as traps and adsorption sites for charge carriers, preventing their recombination. A higher oxygen vacancy content typically results in more pronounced effects [16,39]. Wu et al. [40] utilized UV irradiation to prepare oxygen-vacancy-rich CeO_2_, with increased oxygen vacancy content promoting carrier separation and increasing quantum lifetimes. The photocatalytic reduction of CO_2_ was achieved with an efficiency of 87.4 μmol g^−1^ h^−1^, which is twenty times higher than that of the sample without UV treatment. Grabchenko et al. [41] used various methods to prepare Ag/CeO_2_ photocatalysts, and all the resulting Ag/CeO_2_ samples showed an increase in oxygen vacancy concentration compared to pure CeO_2_, leading to improved catalytic activity in the oxidation of CO and particulate matter.

**Figure 7:**
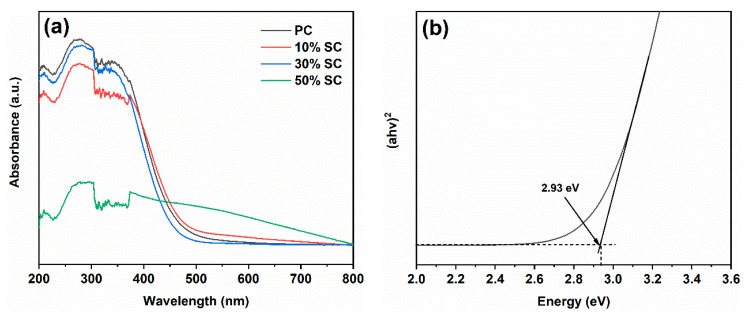
UV–visible absorption spectra (**a**) and band gap of PC (**b**).

Figure 7 depicts the UV–Visible absorption spectra (a) and bandgap width (b) of the samples. PC exhibits strong absorption in the ultraviolet region, with a decrease in light absorption when the wavelength exceeds 400 nm, resulting in less visible light absorption. After the addition of SnO_2_, there was a reduction in UV absorption in the samples. Notably, 50% SC shows the lowest level of UV absorption but a significant increase in visible light absorption. This may be because the SnO_2_ covering the surface of CeO_2_ reduces UV light absorption, while the formation of a heterojunction between the two materials when they come into contact reduces their bandgap width, leading to increased visible light absorption [23]. The bandgap width of PC was calculated to be 2.93 eV.

**Figure 8:**
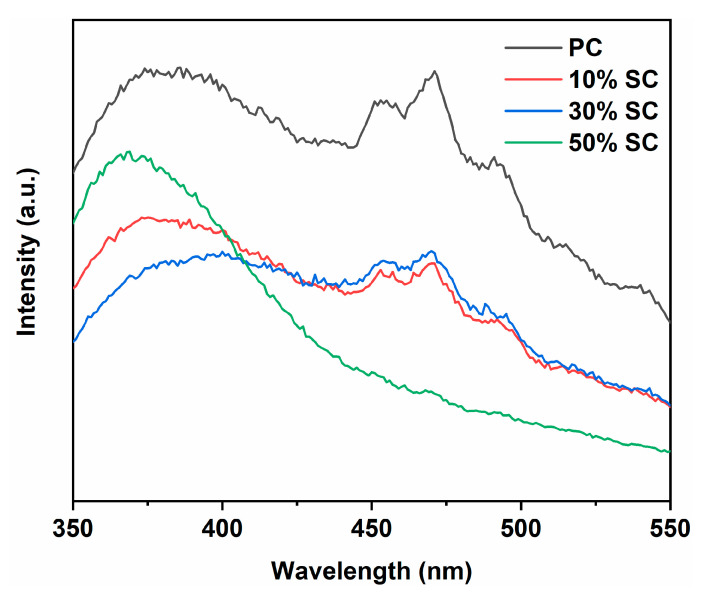
Photoluminescence spectra of samples.

The recombination of photogenerated electrons and holes has a significant impact on the performance of photocatalytic materials. The smaller the number of recombination instances, the greater the number of available carriers available, and this state is advantageous for generating more free radicals. When photo-generated electrons and holes recombine, photons are emitted, giving rise to a peak in photoluminescence (PL). The higher the intensity of the peak, the greater the amount of recombination that occurs. Figure 8 shows the photoluminescence spectra of the samples. PC exhibits the highest peak intensity, and after the addition of SnO_2_, the recombination rate of photogenerated electrons and holes in the samples decreases, indicating that SnO_2_ coupling is beneficial for suppressing the recombination of photogenerated electrons and holes and enhancing quantum efficiency. This may be because the conduction and valence band positions of SnO_2_ and CeO_2_ are different. Photogenerated electrons transfer from the conduction band position of one semiconductor (closer to the top) to that of another semiconductor, while photogenerated holes transfer from the valence band position of one semiconductor (closer to the bottom) to that of another semiconductor, thus suppressing the recombination of photogenerated electrons and holes. Noticeable diffraction peaks appear in the 450–470 nm region, possibly due to photoluminescence resulting from the recombination of photogenerated electrons in oxygen vacancies or crystal defects. It is worth noting that 50% SC has the lowest peak intensity in this region, indicating that the material’s surface has more uncombined oxygen vacancies, a finding that is consistent with the XPS results [42,43,44].

**Figure 9:**
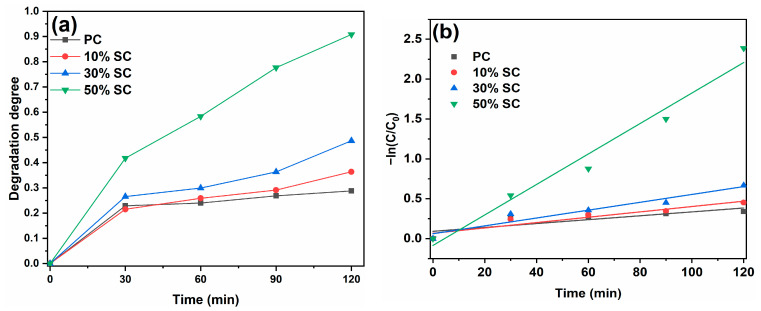
Photodegradation curves (**a**) and kinetics fitting curves (**b**) of samples.

The photocatalytic performance of the samples was evaluated based on the degradation degree of MB, and the degradation degrees of MB as well as the first-order reaction kinetic curves are shown in Figure 9. In Figure 9a, it is evident that after 120 min of illumination, PC achieved a degradation degree of 28.8% with respect to MB, while 10% SC, 30% SC, and 50% SC achieved degradation degrees of 36.8%, 48.7%, and 90.8%, respectively. The coupling of SnO_2_ with CeO_2_ resulted in improved degradation degrees of MB, indicating that SnO_2_ coupling can enhance the photocatalytic performance of CeO_2_. Moreover, the higher the Sn/Ce molar ratio, the more significant the improvement.

The degradation of organic pollutants on the surface of a photocatalyst follows a first-order reaction model: ln(C_0_/C_t_) = kKt = k_app_ × t. Here, C_0_ is the initial absorbance of the pollutant, C_t_ is the absorbance at time t, k is the physical constant of the reaction system, K is the photodegradation degree constant of the reactant, t is the reaction time, and k_app_ is the first-order reaction rate constant [2]. The first-order reaction rate constants (k_app_) for PC, 10% SC, 30% SC, and 50% SC are 0.002 min^−1^, 0.003 min^−1^, 0.005 min^−1^, and 0.019 min^−1^, respectively, which are consistent with the photocatalytic degradation curves. The highest first-order reaction rate constant was observed for 50% SC, which could be attributed to its lower recombination rate of photogenerated charges, with defects like oxygen vacancies, and its higher oxygen vacancy content, resulting in its optimal photocatalytic performance.

**Figure 10:**
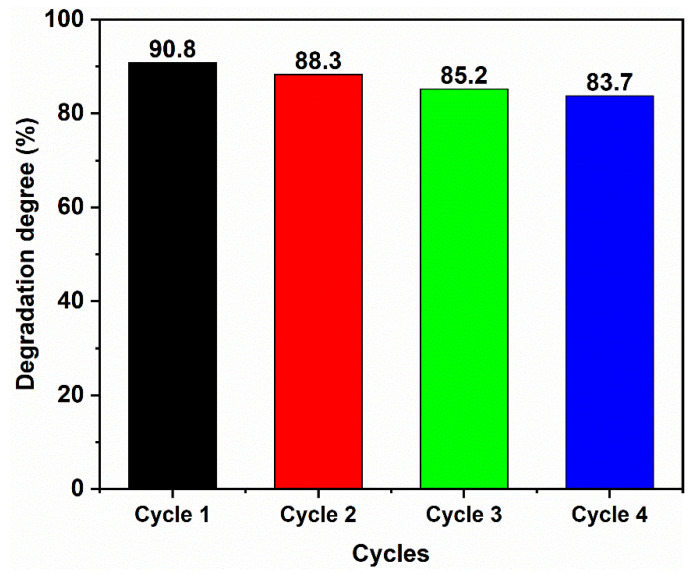
The cyclic experiment of 50% SC photocatalyst for MB degradation.

To investigate the stability of the 50% SC sample, cyclic experiments were performed, and the results are shown in Figure 10. According to the figure, even after four cycles of degradation, the degradation efficiency towards MB remained 83.7%. This indicates that the photocatalytic stability of the sample was relatively high.

Table 1 summarizes the degradation degrees of the CeO_2_-based photocatalytic materials towards MB as shown in the literature, from which it can be seen that the 50% SC obtained in the present work demonstrates considerable photocatalytic activity.

**Figure 11:**
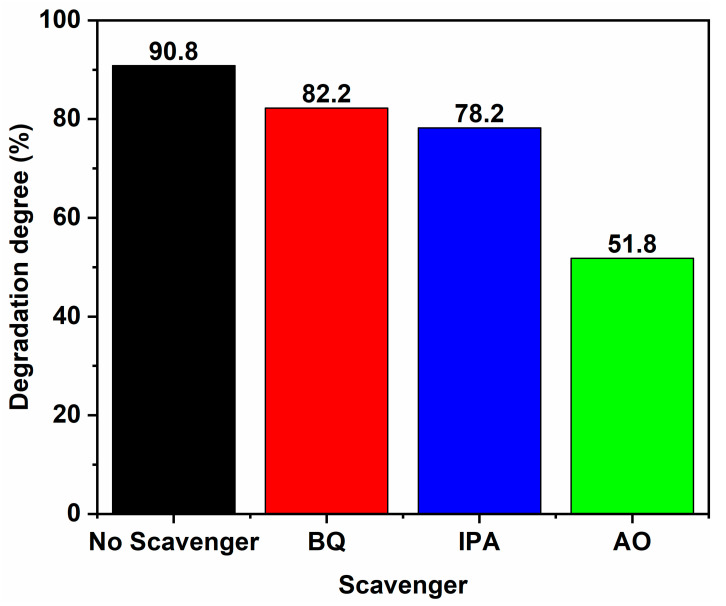
Degradation degrees of 50% SC in the presence of different trapping agents.

The results of the active species experiment for 50% SC are presented in Figure 11. Upon adding BQ, IPA, and AO, the degradation degree of MB in the sample decreased, with the most significant decrease observed when AO was added, reducing the degradation degree from 90.8% to 51.8%. This suggests that h^+^ is the main active species in the photocatalytic process. Additionally, the degradation degrees of the sample decreased from 90.8% to 82.2% and 78.2% when BQ and IPA were added, respectively, indicating the participation of superoxide radicals ·O_2_^−^ and hydroxyl radicals ·OH in the photocatalytic degradation.

**Figure 12:**
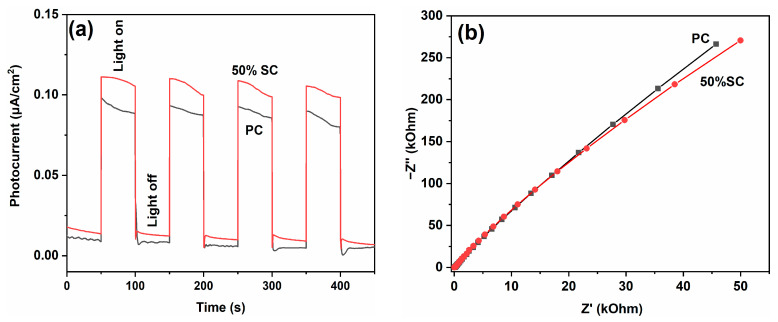
Photocurrents (**a**) and EIS spectra (**b**) of PC and 50% SC.

Electrochemical tests were conducted on PC and 50% SC, and the results are shown in Figure 12. When light shines on the surface of a material, photons interact with electrons in the corresponding sample. The free electrons in the sample can be excited to higher energy levels by photons, forming excited-state electrons. These excited-state electrons can move inside the material and generate a current due to the electric field within the material or an externally applied voltage. By introducing electrodes and an external circuit, it is possible to collect and measure the current generated by the excited-state electrons. The photocurrent density of 50% SC is higher than that of PC, indicating that 50% SC has a higher charge separation efficiency for photogenerated electrons and holes. The Nyquist diameter of 50% SC is smaller than that of PC, suggesting lower resistance to charge transfer during photogenerated charge transfer in 50% SC, resulting in higher charge transfer efficiency [51,52,53]. The electrochemical test results indicate that SnO_2_ coupling can promote the separation and transfer of photogenerated electrons and holes in CeO_2_, improving quantum efficiency. Additionally, the PL results indicate that SnO_2_ coupling can inhibit the recombination of photogenerated electrons and holes in CeO_2_, further enhancing photocatalytic performance.

**Figure 13:**
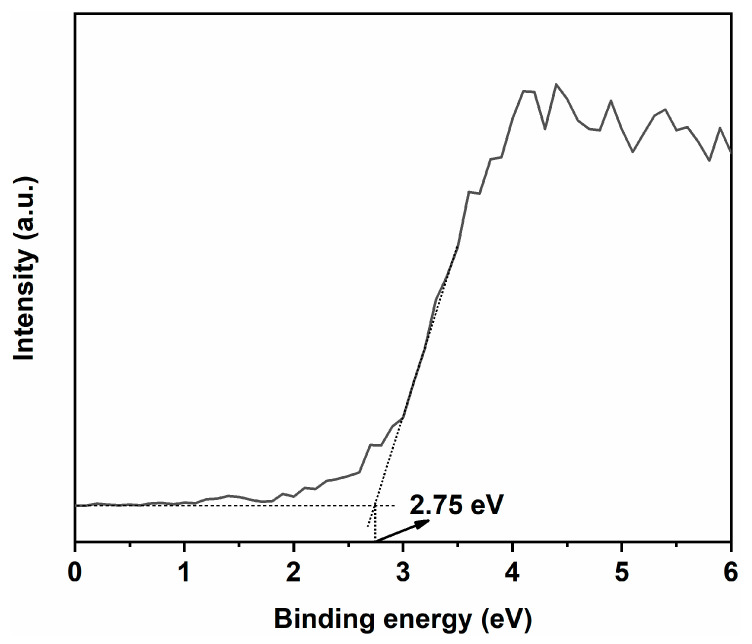
XPS valence band spectrum of CeO_2_.

To determine the valence band position of PC, XPS valence band spectra were obtained, as shown in Figure 13. The valence band position of PC was found to be 2.75 eV. Using the formula E_NEH_ = Φ + E_VL_/eV − 4.44 to calculate the valence band position under standard hydrogen electrode (SHE) conditions, where E_NEH_ is the SHE potential, Φ is the work function of the instrument (4.30 eV), and E_VL_ is the valence band position measured under vacuum conditions [53,54,55], the valence band position of PC under SHE conditions was obtained, amounting to 2.61 eV. Combined with the bandgap width of 2.93 eV for PC, its conduction band position was determined to be −0.32 eV. For PS, the valence band position was 3.66 eV, and the bandgap width was 3.80 eV, so its conduction band position was −0.14 eV [56].

**Figure 14:**
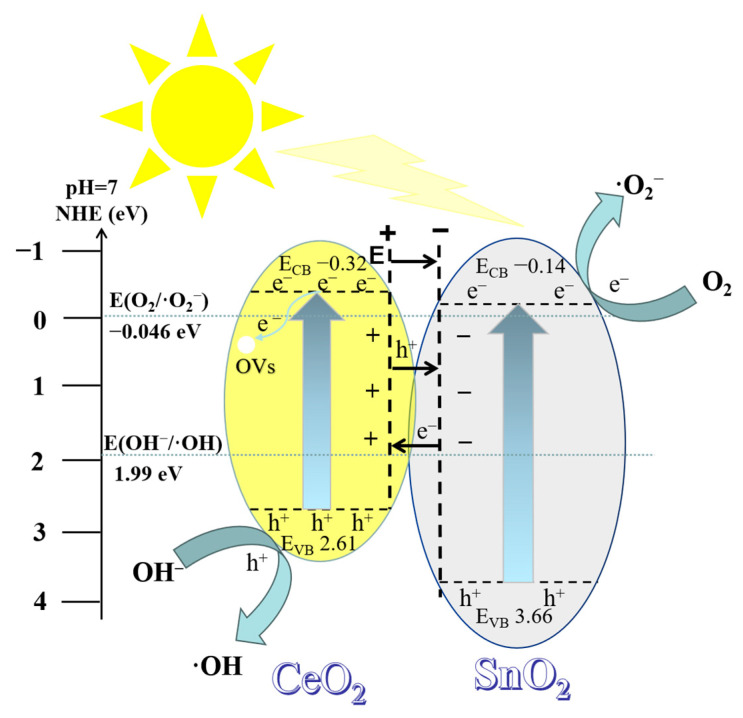
Schematic diagram of charge transfer and free radical formation via light in 50% SC.

Based on the valence band and conduction band potentials of PC and PS, as well as the results of active free radical detection, a schematic diagram of photoinduced charge transfer and free radical generation for 50% SC is presented in Figure 14. Both semiconductor composites could be either Type II or Z-scheme semiconductors. We believe that if charge transfer in 50% SC occurred via a Z-scheme semiconductor mechanism, the electrons in the SnO_2_ conduction band would directly recombine with the holes in the CeO_2_ valence band, leading to a significant consumption of photogenerated holes in the CeO_2_ sample. According to experiments on active species, h^+^ is the primary active species, suggesting that charge transfer in the sample is unlikely to occur via the Z-scheme semiconductor mechanism. On the other hand, when charge transfer occurs via a Type II semiconductor mechanism, several phenomena take place. First, band alignment between the two semiconductors occurs. The bottom energy level of the CeO_2_ conduction band is higher than that of the SnO_2_ conduction band, while the top energy level of the valence band is relatively lower. This band alignment results in the formation of a positive charge in CeO_2_’s conduction band and a negative charge in SnO_2_’s valence band, creating an internal electric field pointing from CeO_2_ to SnO_2_. When 50% SC is exposed to light with an energy greater than 2.93 eV, electrons are excited and thus move from the valence band (VB) of CeO_2_ to the conduction band (CB) of CeO_2_, producing photogenerated electrons (e^−^) with higher activity. Concurrently, photogenerated holes (h^+^) are created in the valence band, and under the influence of the internal electric field, photogenerated electrons and holes move separately towards CeO_2_ and SnO_2_, respectively. The conduction band potential (E_CB_) of 50% SC (−0.14 eV) is lower (more negative) than the electrode potential for the reaction of O_2_ with electrons that leads to the formation of ·O_2_^−^ (O_2_/·O_2_^−^ −0.046 eV), indicating that the conduction band electrons have sufficient reducing power to reduce O_2_ to ·O_2_^−^: O_2_ + e^−^ → ·O_2_^−^. Additionally, the valence band potential (E_VB_) of 50% SC (2.61 eV) is higher (more positive) than the potential for the reaction of valence band holes (h^+^) with OH^−^ that leads to the formation of ·OH (OH^−^/·OH 1.99 eV) [57,58,59,60], suggesting that some valence band holes have sufficient oxidative power to oxidize OH^−^ to ·OH radicals, following the reaction OH^−^ + h^+^ → ·OH. From experiments with active species, it is evident that ·O_2_^−^ and ·OH play a secondary role in the photodegradation of MB, indicating that there are more unreacted h^+^ species. This is because some valence band holes in SnO_2_ transfer to the valence band of CeO_2_, resulting in only a portion of the holes reacting with OH^−^ and thus generating ·OH, while the remaining h^+^ species participate in the photodegradation reaction. In photocatalytic reactions, photogenerated electrons and holes are key intermediates. When photogenerated electrons are excited and thus move to the conduction band, oxygen vacancies can capture these electrons, preventing them from recombining with holes, thus promoting the separation of photogenerated electrons and holes [16,17]. More photo-generated charges can migrate to the surface of the material, and photogenerated charges on the material surface have more opportunities to interact with oxygen and hydroxyl groups, participating in photocatalytic reactions.

## 3. Materials and Methods

First, 10.85 g of cerium nitrate was dissolved in a certain amount of deionized water to prepare 100 mL of solution A. Subsequently, 7.21 g of citric acid was weighed, and while stirring solution A, citric acid was slowly added to it to prepare a mixed solution B. Solution B was placed on a magnetic stirrer and stirred for one hour to ensure complete dissolution. After aging and gelling, solution B was then placed in a drying oven at 100 °C for 10 h to obtain pure CeO_2_, labeled as PC. Additionally, according to Sn/Ce molar ratios of 10%, 30%, and 50%, precisely measured amounts of 0.88 g, 2.63 g, and 4.38 g of SnCl_4_·5H_2_O, respectively, were weighed and slowly added to solution A while stirring. The subsequent steps were the same as above, resulting in SnO_2_ coupled with CeO_2_ samples. The samples with 10%, 30%, and 50% SnO_2_ content were labeled as 10% SC, 30% SC, and 50% SC. To prepare pure tin dioxide, labeled as PS, 3.50 g of SnCl_4_·5H_2_O was dissolved in deionized water to make up 100 mL of solution C. Subsequently, 7.21 g of citric acid was added, and after thorough stirring, solution D was prepared. After aging and gelling, solution D was placed in a drying oven at 100 °C for 10 h.

The crystal structures and compositions of the samples were analyzed using an X-ray diffractometer (XRD), specifically a DX-2700. Additionally, a Hitachi SU8220 scanning electron microscope (SEM) was used for further characterization. A specific surface area measurement was performed using an ASAP2460 analyzer based on the Brunauer–Emmett–Teller (BET) method. The samples’ microscopic morphologies were examined using a JEM-F200 transmission electron microscope (TEM) and a high-resolution transmission electron microscope (HRTEM). The samples’ elemental compositions and chemical states were analyzed using the XSAM800 multifunctional surface analysis system (XPS). Photoluminescence (PL) spectra were measured using an F-4600 fluorescence spectrophotometer equipped with a 320 nm laser wavelength Xenon lamp. For the analysis of light absorption properties, a UV-3600 UV–Visible spectrophotometer was used. Electrochemical performance was assessed using a Chinese DH7000 electrochemical workstation.

The photocatalytic performance of the samples was evaluated based on the degradation degree of the MB solution. In total, 0.1 g of the sample was added to 100 mL of an MB solution and stirred. A Xe lamp was used as the light source, and samples were taken every 30 min. The centrifuged samples were then tested for absorbance at a wavelength of 664 nm, and the degradation degree was calculated using the following formula: (A_0_ − A_t_)/A_0_ × 100%. In addition to the MB degradation system, 2 mL portions of 0.1 mol/L solutions of p-benzoquinone (BQ, ·O_2_^−^ scavenger), isopropanol (IPA, ·OH scavenger), and ammonium oxalate (AO, h^+^ scavenger) were separately added to investigate the active functional groups.

## 4. Conclusions

Pure CeO_2_ was prepared using the sol–gel method, and on this basis, SnO_2_ was successfully coupled to synthesize a SnO_2_-CeO_2_ composite material rich in oxygen vacancies. The results indicate that the specific surface area of the SnO_2_-coupled sample is larger than that of pure CeO_2_, suggesting that the addition of SnO_2_ can enhance the separation and transfer of photogenerated charges while also suppressing their recombination. The presence of oxygen vacancies further promoted the separation of photogenerated charges. The photocatalytic performance of the samples was evaluated using MB as a test pollutant. All the composite samples exhibited improved photocatalytic performance compared to pure CeO_2_, with 50% SC showing the best performance. After 120 min of illumination, 50% SC achieved a degradation degree of 90.8% for MB, demonstrating its excellent photocatalytic activity. In this study, by introducing oxygen vacancies through high-temperature calcination on the foundation of semiconductor-coupled cerium dioxide’s conduction band, the sample material’s photocatalytic performance was significantly enhanced. The introduction of oxygen vacancies altered the material’s microstructure, strengthening its ability to separate photogenerated charge carriers. This improvement in charge separation efficiency led to increased photocatalytic reaction efficiency. This study provides a reference for the preparation of oxygen-deficient type II semiconductor structures. Photocatalytic materials based on cerium dioxide hold promise as highly efficient and environmentally friendly solutions. They have the potential to make significant contributions to controlling water pollution and achieving the sustainable utilization of clean water resources.

## Figures and Tables

**Table 1:** Summary of MB degradation degrees of CeO_2_ reported in the literature.

Reference	Method	Samples	Light Source	Maximum Efficiency
[45]	coprecipitation	Co-CeO_2_	visible-light lamp	96.0% in 360 min
[46]	hydrothermal	Ag-CeO_2_	tungsten lamp (500 W)	94.4% in 100 min
[47]	precipitation	CeO_2_	UV lamp (12 W)	97.4% in 180 min
[48]	ultrasonic-chemical-assisted	CeO_2_	UV lamp (30 W)	85.0% in 75 min
[49]	hydrothermal	ZnO-CeO_2_	xenon lamp (35 W)	85.1% in 120 min
[50]	coprecipitation	Nb-CeO_2_	mercury lamp (125 W)	96.8% in 300 min
This work	sol–gel	SnO_2_-CeO_2_	Xe lamp (250 W)	90.8% in 120 min

## Data Availability

Not applicable.
